# Could a careful clinical examination distinguish physiologic phimosis from balanitis xerotica obliterans in children?

**DOI:** 10.1007/s00431-020-03881-4

**Published:** 2020-11-23

**Authors:** Filippo Ghidini, Calogero Virgone, Rebecca Pulvirenti, Emanuele Trovalusci, Piergiorgio Gamba

**Affiliations:** Pediatric Surgery Unit, Department of Women’s and Children’s Health, Padua General Hospital, Padua University, via Nicolò Giustiniani, 35121 Padua, Italy

**Keywords:** Balanitis xerotica obliterans, Lichen sclerosus, Children, Preputial histology, Phimosis

## Abstract

The diagnosis of balanitis xerotica obliterans (BXO) in children may be challenging, since clinical examination only could lead to an underestimation of its incidence. The aim of this retrospective and single-centre study is to assess the diagnostic performance of clinical examination, together with clinical history, in identifying BXO. Ninety-seven children underwent circumcision for phimosis from 2015 to 2019. Histology was routinely performed. Cohen’s kappa coefficient, sensitivity, specificity, predictive values, likelihood ratios and accuracy of macroscopic appearance of the foreskin, steroid administration and past medical history were estimated. Forty-eight patients (50%) were affected by BXO; 31 of them (69%) presented with suggestive clinical signs. A strictured or whitish urethral meatus was detected during surgery in nine cases (19%); this was associated to allergic or immune diseases (*p* = 0.046). Foreskin appearance alone mildly correlated with histology (*k* = 0.494; *p* < 0.001) and it showed a diagnostic accuracy of 75%. The specificity and positive predictive value of abnormal macroscopic findings at examination, together with a positive clinical history for other allergic or immune diseases, and/or for balanitis, were 100% and the positive likelihood ratio was greater than 10. Conversely, sensitivity decreased to 4.5% (95% CI 0–11%).

*Conclusion*: Foreskin appearance together with clinical history could predict BXO with certainty. However, since the absence of a positive medical history could not exclude the diagnosis, foreskin histology is still highly recommended.

**Table:** 

**What is Known:** *• Occurrence of balanitis xerotica obliterans may be underestimated in children and it could lead to long-term complications.* *• The diagnostic accuracy of clinical examination is controversial.*	
**What is New:** *• Clinical signs together with patients’ medical history present high specificity and positive predictive values but low sensitivity.* *• When suggestive clinical aspects are present, patient should be referred to surgery avoiding prolonged conservative treatment; and preputial histology is highly recommended.*

**Supplementary Information:**

The online version contains supplementary material available at 10.1007/s00431-020-03881-4.

## Introduction

Non-retractable prepuce or phimosis affects up to 50% of school-age boys [1]. This finding is due to a physiological process. However, it affects up to 20% of circumcised children [2]. Phimosis is caused by balanitis xerotica obliterans (BXO), also known as lichen sclerosus (LS). BXO is defined as chronic inflammatory dermatitis with an unknown aetiology involving not only the foreskin but also the glans and the urethral meatus, possibly leading to meatal stenosis and urinary symptoms in children [3].

On clinical examination, a whitish scar ring around the foreskin, together with white plaques on the glans, is often found. However, the diagnosis should be confirmed by histology, since the macroscopic appearance may not be sufficient to correctly identify BXO, and the actual correlation rate of this with a positive histology is unknown. Microscopically, BXO affects almost exclusively the inner mucosal surface of the prepuce and is characterised by the presence of a subepidermal inflammatory layer composed of homogenised collagen and lymphocytes [4].

The most commonly accepted treatment consists of circumcision [5]. In recent years, preputioplasty has been demonstrated to be an effective surgical option when parents prefer sparing the foreskin because of their social and cultural background, especially in Western countries [6]. Moreover, the long-term efficacy of topical steroids has not been proven so far [7].

The aim of this study was to assess the correlation of the macroscopic appearance of the foreskin, alone or in combination with other clinical features (such as previous infections, association with autoimmune disease, and lack of improvement after local steroid treatment), with the histological findings after circumcision. Furthermore, the diagnostic accuracy of the clinical features in predicting the presence of BXO was estimated to help physicians indicate a surgical procedure and properly counsel patients and parents when a surgical procedure is indicated.

## Materials and methods

### Population

This was a single-centre and retrospective study. The inclusion criteria consisted of the presence of phimosis as the primary indication for circumcision, the absence of any associated penile malformation and an available foreskin pathology result (at our centre, histology is routinely performed during all non-ritual circumcisions).

Demographic data (including ethnicity) and clinical data regarding boys undergoing consultation and surgery for phimosis from January 2015 to December 2019 were reviewed. Comorbidities (atopic dermatitis, bronchial asthma, celiac disease, and thyroid autoimmune disease), surgical reports, pathology results, topical steroid treatment and other clinical details, including the appearance of the urethral meatus during surgery, were included.

A senior paediatric surgeon performed the initial clinical assessment in all patients. Whitish scars or atrophic plaques on the distal foreskin, when found on physical examination, were considered suggestive of BXO. Topical steroids were administered 2 weeks per month for a maximum of three cycles when a non-retractable prepuce without macroscopic signs of BXO was observed. In some patients with a clinical suspicion of BXO, topical steroids were prescribed to avoid potential complications, such as urinary retention, before circumcision. Circumcision was indicated in children aged more than 6 years. Special consideration was given to repeated episodes of infection (> 2/year), such as balanitis and lower urinary tract infections, or to a lack of improvement after multiple cycles (> 2/year) of topical steroids.

The external meatus was defined to be macroscopically abnormal when, during the surgery, presented with a whitish appearance or a stricture (defined as difficulty passing an 8–10 French probe or catheter after having removed the adherences of the foreskin).

If the diagnosis of BXO was confirmed, the boy was referred to a dermatologist, and screening for thyroid and celiac disease was advised, since cutaneous involvement and association with these autoimmune diseases have been described in the literature, especially in adulthood [8, 9]. All the reported complications were classified according to the modified Clavien-Dindo scale [10]. Adverse events were defined as surgical site infections (SSIs), wound leaks and anomalies (such as granulomas or delayed healing of surgical wounds, which was estimated as 30 days) [11].

### Statistical analysis

Categorical variables are reported as numbers and frequencies (%), and continuous variables are reported as medians and interquartile ranges (IQRs). The overall population was divided into two groups: patients with a histological diagnosis of BXO (group A) and those without BXO (group B).

The inter-reliability between the clinical and histological features of BXO was estimated according to Cohen’s kappa coefficient (*k*). The sensitivity, specificity, positive predictive value (PPV), negative predictive value (NPV), negative likelihood ratio (LR−), positive likelihood ratio (LR+) and accuracy for the macroscopic signs and for different combinations with other clinical features were estimated.

The aim was to identify the clinical features with the best diagnostic performance. A *p* value ≤ 0.05 was considered statistically significant, and the 95% confidence intervals are reported.

IBM® SPSS Inc., version 26.0, was used for the statistical analysis.

## Results

### Patients

Ninety-seven children met the inclusion criteria in the timeframe of this study. Data regarding the macroscopic appearance of the foreskin, infections before surgery and topical steroids were missing for six (6.2%), four (4.1%) and two (2.1%) patients, respectively.

The median age at circumcision was 9.0 years (IQR 6.6–11 years), and the median body weight was 31 kg (20–39 kg).

Forty boys (44%) presented with clinical features consistent with BXO, and topical steroids were administered to 25 of them (63%). The majority of the boys with a normal physical examination (37/51, 73%) received topical steroids after surgical consultation.

In 10 patients (10%), a whitish or strictured urethral meatus was found at the time of circumcision. The histological examination was diagnostic for BXO in 48 patients (50%). Fifteen grade 2 complications were noted in 16% of children: eight experienced delayed wound healing (9.3%), four experienced wound leaks (4.1%) and two experienced SSIs (2.1%). Reoperations were not necessary in any patient.

Forty-three boys (44%) were affected by other diseases, most of which (35/43) re-included in the atopic spectrum (e.g. dermatitis and bronchial asthma).

Additionally, details on the overall cohort can be found in the Appendix (Table A).

### Differences between the two groups

The median age at the first surgical consultation (9.7 years; IQR 6.5–11 years) and age at intervention (10 years; IQR 7.6–12 years) were higher in the BXO group than in the non-BXO group (*p* = 0.006; *p* = 0.030). No difference was found regarding the interval before surgery (*p* = 0.069).

Clinical features suggestive of BXO were present in 31 affected patients (69%), and this rate was greater than that in unaffected patients (*p* < 0.001).

Furthermore, the BXO group presented nine anomalies of the urethral meatus (90%) (*p* = 0.007). No other differences were found between the two groups, as shown in Table 1.

**Table 1:** Comparison between the patients affected by BXO and those not affected

	Patients with BXO (*n* = 48)	Patients without BXO (*n* = 49)	*p* value
Age at consultation (years; median, IQR)	9.7 (6.5–11)	6.8 (3.9–9.2)	.006
Caucasian ethnicity (*n*, %)	45 (94)	43 (88)	.309
Comorbidity (*n*, %)	23 (48)	20 (41)	.482
Age at circumcision (years; median, IQR)	10 (7.6–12)	7.6 (5.3–11)	.030
Body weight (kg, median, IQR)	33 (23–40)	28 (19–38)	.108
Signs of BXO* (*n*, %)	31(69)	9 (20)	< .001
At least one episode of infections before surgery (balanitis, low UTIs)* (*n*, %)	11 (23)	4 (8.7)	0.54
No improvement after topical steroid treatment* (*n*, %)	30 (63)	35 (75)	.210
Abnormal urethral meatus at intervention (*n*, %)	9 (19)	1 (2.0)	.007
Complications after circumcision (*n*, %)	9 (19)	6 (12)	.376

*Missing data

None of the patients affected by BXO reported lower urinary tract symptoms or underwent further procedures for meatal involvement after circumcision.

### Clinical diagnosis of BXO

The presence of suggestive clinical signs showed a mild correlation with the histological diagnosis of BXO (*k* = 0.494; *p* < 0.001), even though clinical signs did not correlate with involvement of the urethral meatus (*k* = − 0.019; *p* = 1.000).

The sensitivity and specificity of clinical signs were 69% (95% CI 55–82%) and 80% (95% CI 69–92%), respectively. The PPV and NPV were 78% (95% CI 65–90%) and 28% (95% CI 15–40%), respectively, with a diagnostic accuracy reaching 75%.

Table 2 reports the diagnostic performance of the clinical signs and of the following preoperative factors: presence of autoimmune or allergic diseases, having experienced at least one episode of balanitis or a lower UTI and the lack of improvement after topical steroids. When other preoperative factors were considered together with clinical signs, the specificity and PPV increased up to 100%; conversely, the sensitivity and diagnostic accuracy decreased considerably.

**Table 2:** Diagnostic accuracy of the clinical variables

	Sensitivity (%, 95 CI)	Specificity (%, 95 CI)	PPV (%, 95 CI)	NPV (%, 95 CI)	LR+	LR−	Accuracy (%)
Signs	69 (55–82)	80 (69–92)	78 (65–90)	28 (15–40)	3.52	0.39	74.7
Signs + comorbidity	31 (19–45)	93 (86–100)	82 (64–100)	42 (31–53)	4.78	0.74	62.6
Signs + comorbidity + infections	4.5 (0.0–11)	100	100	49 (38–59)	> 10	0.96	52.3
Signs + comorbidity + infections + no improvement	4.3 (0.0–10)	100	100	50 (40–60)	> 10	0.96	51.1

*IQR*, interquartile range; *PPV*, positive predictive value; *NPV*, negative predictive value; *LR+*, positive likelihood ratio; *LR−*, negative likelihood ratio

The likelihood ratios were also calculated: clinical signs together with other preoperative factors showed a positive likelihood ratio greater than 10, with a negative likelihood ratio of 1.0.

## Discussion

In our cohort, almost half of the circumcised patients had histologically proven BXO. These children were older, and most of them presented with suggestive symptoms. An abnormal urethral meatus was encountered in 19% of patients during surgery. These clinical findings mildly correlated with the histological diagnosis of BXO. However, when comorbidity and episodes of infection were considered together with the macroscopic appearance, the specificity and PPV increased up to 100%, whereas the sensitivity was extremely low.

Although the rate of phimosis caused by BXO in our population may be considered high, it is in line with that reported in the literature, as a recent systematic review by Li et al. found an incidence ranging from 2 to 95% among circumcised children [12].

The aetiology of BXO is multi-factorial, as in several chronic inflammatory diseases, and the association with other inflammatory or allergic diseases is reported to be relevant [13–15]. We found that the presence of comorbidities was associated with an increased risk of meatal involvement. We therefore speculate that comorbidity could account for the severity of BXO, but Homer et al. reported that up to 20% of patients might need a meatal procedure after circumcision and did not identify any predictive factor [16].

The diagnosis of BXO cannot rely only on the macroscopic appearance of the foreskin or symptoms, since diagnosis based on a clinical examination could underestimate its occurrence in up to 50% of cases [12, 17]. However, a large published series estimated the diagnostic accuracy of an abnormal preputial appearance to be more than 80% [11, 18] despite reports claiming that the initial stages of BXO might not show peculiar findings on clinical examination [19].

In our series, children affected by BXO were older than those with physiological phimosis, confirming that the epidemiological distribution of BXO could be age-dependent [20, 21]. Patients with BXO may present with higher rates of abnormal clinical findings than the general population. This result could be undermined by the parental desire to avoid circumcision [6], especially if their child complained of mild symptoms at the initial surgical consultation. In the first instance, the surgeon himself may have preferred a conservative approach, postponing circumcision after the failure of multiple cycles of steroid treatment [22]. Notwithstanding, the interval before circumcision did not vary between the two groups.

This report underlines how clinical features, together with an accurate medical history, may be helpful in identifying children who may need circumcision, which is considered the definitive treatment for BXO [23]. Furthermore, the high positive likelihood ratio may suggest a high pre-test probability of disease. This diagnostic approach could avoid inefficacious and prolonged courses of steroid application and a delay in surgical treatment.

Clinical signs together with a medical history considerably decreased the sensitivity, but they could exclude BXO with certainty. Hence, a histological examination is highly recommended to avoid misdiagnoses, which could expose patients to a wide spectrum of long-term complications, from lower urinary tract symptoms and meatal stenosis to penile cancer in adulthood [24].

Parents and patients should therefore be informed about the diagnostic process, which might rely not only on a physical examination but also on surgical procedures to obtain a histological sample of the foreskin. Moreover, the high specificity of clinical findings may help surgeons identify populations that could require careful follow-up after surgery.

The limitations of this study were its retrospective and single-population design, which could influence data collection, the reporting of clinical features and the true incidence of BXO. Larger prospective cohorts may validate these findings.

In conclusion, the presence of BXO among children affected by phimosis should not be underestimated since the macroscopic appearance of the foreskin mildly correlated with the histological results. Nonetheless, clinical features, together with the aforementioned comorbidities and a history of repeated infections, may be considered reliable when raising the suspicion of BXO. These considerations make preputial histology highly recommended, even in patients with a low clinical suspicion.

## Supplementary information

ESM 1 (DOCX 14 kb)

## Data Availability

Data are not stored publicly due to privacy restrictions.

## Abbreviations

BXO: Balanitis xerotica obliterans
UTI: Urinary tract infection
SSI: Surgical site infection
IQR: Interquartile range
PPV: Positive predictive value
NPV: Negative predictive value
LR+: Positive likelihood ratio.
LR−: Negative likelihood ratio

## References

[1] Hsieh TF, Chang CH, Chang SS (2006). Foreskin development before adolescence in 2149 school-boys. Int J Urol.

[2] Sneppen I, Thorup J (2016). Foreskin morbidity in uncircumcised males. Pediatrics.

[3] McLelland J (2004). Lichen sclerosus in children. J Obstet Gynaecol.

[4] Singh L, Sengar M, Goyal S, Mansi M, Khurana N, Mohta A (2018). Childhood phimosis secondary to lichen sclerosus: is there a spatial pattern of histopathological changes?. Am J Dermatopathol.

[5] World Health Organization, Department of Reproductive Health and Research and Joint United Nations Programme on HIV/AIDS (UNAIDS). Male circumcision: global trends and determinants of prevalence, safety and acceptability. Available from: https://www.who.int/reproductivehealth/publications. Accessed 24 July 2020

[6] Wilkinson DJ, Lansdale N, Everitt LH, Marven SS, Walker J, Shawis RN, Roberts JP, Mackinnon AE, Godbole PP (2012). Foreskin preputioplasty and intralesional triamcinolone: a valid alternative to circumcision for balanitis xerotica obliterans. J Pediatr Surg.

[7] Tong LX, Sun GS, Teng JM (2015). Pediatric lichen sclerosus: a review of the epidemiology and treatment options. Pediatr Dermatol.

[8] Jacobs L, Gilliam A, Khavari N, Bass D (2014). Association between lichen sclerosus and celiac disease: a report of three pediatric cases. Pediatr Dermatol.

[9] Kantere D, Allergien G, Gillstedt M, Pujol-Calderon F, Tunbäck P (2017). Clinical features, complications and autoimmunity in male lichen sclerosus. Acta Derm Venereol.

[10] Mitropoulos D, Artibani W, Biyani CS, Bierggard Jensen J, Rouprêt M, Truss M (2018). Validation of the Clavien-Dindo grading system in urology by the European Association of Urology guidelines ad hoc panel. Eur Urol Focus.

[11] Pradhan A, Patel R, Said AJ, Upadhyaya M (2019). 10 years’ experience in balanitis xerotica obliterans: a single-institution study. Eur J Pediatr Surg.

[12] Li J, Deng C, Peng Q (2018). Underestimation of genital lichen sclerosus incidence in boys with phimosis: results from a systematic review. Pediatr Surg Int.

[13] Becker K, Meissner V, Farwick W, Bauer R, Gaiser MR (2013). Lichen sclerosus and atopy in boys: coincidence or correlation?. Br J Dermatol.

[14] Charlton OA, Smith SD (2019). Balanitis xerotica obliterans: a review of diagnosis and management. Int J Dermatol.

[15] Fuchs ME, Beecroft N, Dajusta DG, McLeod DJ (2017). The association between BXO and obesity in boys undergoing circumcision. Glob Pediatr Health.

[16] Homer L, Buchanan KJ, Nasr B, Losty PD, Corbett HJ (2014). Meatal stenosis in boys following circumcision for lichen sclerosis (balanitis sclerotic obliterans). J Urol.

[17] Kato T, Mizuno K, Nishio H, Moritoki Y, Nakane A, Kurokawa S, Kamisawa H, Maruyama T, Yasui T, Hayashi Y (2018). Can lichen sclerosus be diagnosed by preputial appearance or symptoms?. Res Rep Urol.

[18] Gargollo P, Kozakewich H, Bauer S (2005). Balanitis xerotica obliterans in boys. J Urol.

[19] Boksh K, Patwardhan N (2017). Balanitis xerotica obliterans: has accuracy improved with time?. JRSM Open.

[20] Nelson DM, Peterson AC (2011). Lichen Sclerosus: epidemiological distribution in an equal access health care system. J Urol.

[21] Nguyen ATM, Holland AJA (2020). Balanitis xerotica obliterans: an update for clinicians. Eur J Pediatr.

[22] Metcalfe PD, Elyas R (2010). Foreskin management: survey of Canadian pediatric urologists. Can Fam Physician.

[23] Celis S, Reed F, Murphy F, Adams S, Gillick J, Abdelhafeez AH, Lopez PJ (2014). Balanitis xerotica obliterans in children and adolescents: a literature review and clinical series. J Pediatr Urol.

[24] Bochove-Overgaauw DM, Gelders W, De Vylder AM (2009). Routine biopsies in pediatric circumcision: (non) sense?. J Pediatr Urol.

