# Supplementary material for: Could a careful clinical examination distinguish physiologic phimosis from balanitis xerotica obliterans in children?
Source: Eur J Pediatr. 2020 Nov 23;180(2):591–5. doi: 10.1007/s00431-020-03881-4 (PMC7813725; doi:10.1007/s00431-020-03881-4)
Supplement: Supplementary file 1 — (DOCX 14 kb) [file 431_2020_3881_MOESM1_ESM.docx]

**Appendices**

*Table A. Characteristics of the 97 circumcised patients.*

| **Age at consultation**  *(yrs; median, IQR)* | 8.3 (5.8-11) |
| --- | --- |
| **Caucasian ethnicity**  *(n, %)* | 88 (91) |
| **Comorbidity**  *(n, %)* | 43 (44) |
| **Time between diagnosis and circumcision**  *(mos; median; IQR)* | 4.2 (2.0-9.1) |
| **Age at circumcision**  *(yrs; median, IQR)* | 9.0 (6.6-11) |
| **Body Weight**  *(kg, median, IQR)* | 31 (20-39) |
| **Signs of BXO***  *(n, %)* | 40 (44) |
| **At least one episode of infection before surgery (balanitis, low UTIs)***  *(n, %)* | 15 (16%) |
| **No improvement after topical steroid treatment***  *(n, %)* | 65 (68) |
| **Abnormal urethral meatus at intervention**  *(n, %)* | 10 (10) |
| **Histological diagnosis of BXO**  *(n,%)* | 48 (50) |
| **Complications after circumcision**  *(n,%)* | 15 (16) |

*missing data
